# Season of birth, clinical manifestations and Dexamethasone Suppression Test in unipolar major depression

**DOI:** 10.1186/1744-859X-6-20

**Published:** 2007-08-03

**Authors:** Konstantinos N Fountoulakis, Apostolos Iacovides, Michael Karamouzis, George S Kaprinis, Charalambos Ierodiakonou

**Affiliations:** 1 Third Department of Psychiatry, Aristotle University of Thessaloniki, Greece; 2 Laboratory of Biochemistry, Aristotle University of Thessaloniki, Greece

## Abstract

**Background:**

Reports in the literature suggest that the season of birth might constitute a risk factor for the development of a major psychiatric disorder, possibly because of the effect environmental factors have during the second trimester of gestation. The aim of the current paper was to study the possible relationship of the season of birth and current clinical symptoms in unipolar major depression.

**Methods:**

The study sample included 45 DSM-IV major depressive patients and 90 matched controls. The SCAN v. 2.0, Hamilton Depression Rating Scale (HDRS) and Hamilton Anxiety Scale (HAS) were used to assess symptomatology, and the 1 mg Dexamethasone Suppression Test (DST) was used to subcategorize patients.

**Results:**

Depressed patients as a whole did not show differences in birth season from controls. However, those patients born during the spring manifested higher HDRS while those born during the summer manifested the lowest HAS scores. DST non-suppressors were almost exclusively (90%) likely to be born during autumn and winter. No effect from the season of birth was found concerning the current severity of suicidal ideation or attempts.

**Discussion:**

The current study is the first in this area of research using modern and rigid diagnostic methodology and a biological marker (DST) to categorize patients. Its disadvantages are the lack of data concerning DST in controls and a relatively small size of patient sample. The results confirm the effect of seasonality of birth on patients suffering from specific types of depression.

## Background

The season of birth used to be an important element in the pre-scientific era of medicine [1]. 'Melancholia' was the term ancient Greeks used in order to define what today is called depression, and the word came from the theory of the four fluids of the human body. A further development of this theory, which followed wider philosophical streams, placed the microcosm of man inside the frame of a wider macrocosm, the universe ('kosmos'). In this sense, the border between man and universe could be considered as arbitrary, and man was believed to follow the laws of nature, in a sense radically different from what is accepted today. In this conceptual frame, depression was believed to come from an excess of black bile ('melaina chole') and considered to be an analog of spring. Psychiatry had been dominated by the remnants of these beliefs for more than 2,000 years and, even today, they survive in the lay people's beliefs in astrology and the zodiac.

Schizophrenia is the mental disorder best studied concerning the relation to the patients' season of birth. It is believed to associate with a modest excess of winter births. However, this relation seems to be weak and not specific for schizophrenia, but rather it might also concern other mental disorders [2], and in particular mood disorders [3].

In accord with contemporary theories concerning the etiopathogenesis of mental disorders (according to the biopsychosocial model) that include the influence of environmental factors, especially during gestation or the first months and years of life, various theories have been put forward to explain a possible birth-season effect. These theories include various potential deleterious factors such as temperature, nutritional deficiencies, infectious agents etc., or a genetic factor in those with a propensity for schizophrenia that protects against infection. Generally, the harmful effects hypothesis (i.e., that schizophrenia involves infectious agents) is considered to be more valid [4]. From these factors, only viral epidemics during the winter months seem to apply as a stable worldwide season-related risk factor. Indeed, some authors argue there might be a weak non-specific connection of exposure *in utero *to influenza epidemics to most major mental disorders [2]. There are also studies that report the lack of a birth-season effect; for example, in depression related to Alzheimer's disease [5].

The aim of the present study was to investigate the relationship of current depressive and anxiety symptomatology, as well as current and historical suicide attempts, with the season of birth of unipolar depressive patients. The logic behind this was to test whether environmental factors related to a specific season of the year could play a role in the etiopathogenesis of depression by acting during the gestation period or the first months of life.

## Methods

A total of 135 subjects took part in the study. Of that total, 45 were patients (10 males and 35 females) aged 19–60 years old (mean = 39.3, SD = 12.2) suffering from major depression according to DSM-IV.

A total of 90 of the 135 patients in the study were gender (70 females and 20 males) and age (mean = 38.7, SD = 11.02) matched control subjects, free of any mental or somatic disorder. Matching was performed on an individual basis with a maximum of 2 years difference.

All patients and controls were born in the northern part of Greece. All provided written informed consent.

The definition of 'season' was made according to the calendar (December to February: winter, March to May: spring, June to August: summer, September to November: autumn). The DST samples were collected during all seasons of the year without any systematic bias.

### Patients

All depressed patients were either inpatients or outpatients of the Third Department of Psychiatry, Aristotle University of Thessaloniki, University Hospital AHEPA. All were proven medically healthy when given physical and laboratory examinations, including a routine EEG and a thyroid profile.

The Schedules for Clinical Assessment in Neuropsychiatry (SCAN)v. 2.0 [6] were used to assist clinical diagnosis. However, during the time period that the current study was being written, the software and the algorithms for SCAN were not available, and therefore could not automatically provide the diagnosis. The data collected using SCAN by one interviewer were combined with the clinical assessment of a second interviewer, and the diagnosis was reached by consensus of the two researchers.

The quantification of depressive and anxiety symptomatology was performed with the 17 items Hamilton Depression Rating Scale (HDRS-17) [7] and the Hamilton Anxiety Scale (HAS) [8]. HDRS-17 item no. 3 was used to assess the severity of current suicidal ideation.

On the basis of the semi-structured clinical interview, any recent suicidal attempt was registered as well as any similar attempt at any time point in the past. All attempts were carried out by swallowing pills, and no attempt was 'violent' (i.e., by hanging, the use of firearms, fall from great height etc.).

All depressed patients underwent the Dexamethasone Suppression Test (DST) [9]. According to the DST protocol, 1 mg of dexamethasone was given orally at 23.00 on day 1, and three samples of blood were taken at 23.00 on day 1 and at 16.00 and 23.00 on day 2, in order to measure serum cortisol levels. A subject was considered as DST non-suppressor if either cortisol measure on day 2 was over 5 μg/dl.

No patients fulfilling criteria for catatonic or psychotic features or seasonal pattern were included. No depressed patient manifested seasonal patterns of symptomatology. In all patients, the onset of depressive or anxious symptomatology appeared after the age of 18.

### Controls

Control subjects came from the medical and nursing stuff and their families.

One of the authors performed a clinical interview of each control subject, in order to determine the current medical and psychiatric status. Only subjects normal beyond doubt according to the clinical interview were included in the study. DST was not applied in controls.

It should be noted that is obvious that the methodology differs between patients and controls. However, the task was to identify healthy individuals (this screening can be achieved by clinical interview alone), and to diagnose depression in patients, which can be reliably achieved only with the use of a structured interview. DST non-suppression is seen in about 10% of healthy individuals and it is due to a number of factors such as stress, medication, physiological instability, but also a large proportion is of unknown cause [10]. It is obvious that the lack of DST data in control subjects constitutes a methodological yet unavoidable drawback of the current study.

### Statistical analysis

The statistical analysis [11] included the Chi-square test and one-way ANOVA with LSD as the post-hoc test.

## Results

A total of 10 (22.22%) depressed patients were DST non-suppressors and 35 (77.78%) were suppressors. The distribution of the birth rate in control subjects, depressives, DST suppressors and non-suppressors is shown in Table 1. The chi-square test revealed a significant difference between controls and DST non-suppressors concerning the season of birth (p = 0.011), while the comparison between controls and suppressors revealed no significant differences (p = 0.559). This finding suggests that DST non-suppressor depressives were more likely to have been born during the autumn and winter and less likely during the spring or summer.

**Table 1:** Distribution by season of year of births in the diagnostic groups

**Season**	**Depressives (D), n = 45**	**Controls (C), n = 90**	**DST non-suppressors (NS), n = 10**	**DST suppressors (S), n = 35**
**Winter**	15 (33.33%)	24 (26.67%)	4 (40.00%)	11 (31.43%)
**Spring**	9 (20.00%)	27 (30.00%)	1 (10.00%)	8 (22.86%)
**Summer**	9 (20.00%)	26 (28.89%)	0 (0.00%)	8 (22.86%)
**Autumn**	12 (26.67%)	13 (14.44%)	5 (50.00%)	8 (22.86%)
**Total**	45 (100%)	90 (100%)	10 (100%)	35 (100%)

The mean HDRS-17 score for depressed patients was 24.95 ± 6.23 (range 11–40). The respected HAS score was 33.28 ± 13.74 (range 8–45). One-way ANOVA results, with season of year (4 levels) as the grouping variable and HDRS-17 and HAS scores as dependent variables, suggested an effect of season (p = 0.035). The LSD post-hoc test suggested that there was a significant difference in HDRS-17 scores between winter and spring (23.58 ± 6.61 vs 30.29 ± 6.60, p = 0.025) and between spring and summer (30.29 ± 6.60 vs 21.29 ± 4.68, p = 0.008) and in HAS scores between spring and summer (38.29 ± 6.60 vs 22.00 ± 13.76, p = 0.031).

The mean values and standard deviations of HDRS and HAS scores in the depressed patients by season of birth are shown in Table 2. A significant difference was observed in HDRS score between those born during spring and those born during summer. The impression the data shown in Table 2 gives is that patients born during spring manifested higher scores in both scales, in comparison to all the other patients, but this finding was not significant.

**Table 2:** Means and standard deviation of HDRS-17, HAS and HDRS item no. 3 (suicidal ideation) scores by season of birth

	**Winter**		**Spring**		**Summer**		**Autumn**	
	**Mean**	**SD**	**Mean**	**SD**	**Mean**	**SD**	**Mean**	**SD**

**HDRS**	23.58	6.61	30.29	6.60	21.29	4.68	26.20	5.69
**HAS**	37.08	17.72	38.29	6.60	22.00	13.76	31.30	10.49
**HDRS item no. 3**	1.33	1.23	2.00	1.29	1.86	1.46	2.10	0.99

Nine patients (20%) had recently attempted suicide, and 15 patients (33.33%) had attempted suicide at some time in the past. The chi-square test revealed no effect of the season of birth (p = 0.79 and 0.85 respectively). The distribution of attempts by season of birth, are shown in Table 3. Although the chi-square revealed no significant results, it seems there is a tendency for those patients born during the autumn and winter to manifest a higher rate of suicide attempts.

**Table 3:** Distribution of suicide attempts concerning the season of birth of the patients (no significant differences observed)

	**Recent attempt**		**Attempt at any time in the past**	
	**No**	**Yes**	**No**	**Yes**

**Winter**	12	2	11	4
**Spring**	7	2	6	3
**Summer**	7	3	5	4
**Autumn**	10	2	8	4
**Total**	36	9	30	15

A secondary analysis with one-way ANOVA and season of birth as the grouping variable and HDRS item no. 3 (suicidal ideation) as the dependent variable revealed no significant differences (p = 0.48), although there was a tendency for this item score to be lower for those patients born during the winter period and almost the same for those born during the other three seasons. The respected means and standard deviations are shown in Table 2.

## Discussion

The current study investigated the relationship of current depressive and anxiety symptomatology as well as current and anytime suicide attempts with the season of birth of unipolar depressive patients. There are a limited number of studies in the international literature addressing this question, and to our knowledge the current study is the first that also uses a biological factor (the Dexamethasone Suppression Test) to subcategorize depressive patients. The results suggest that depressed patients born during the spring (March/April/May) manifested higher levels of depressive symptomatology while those born during the summer manifested the lowest anxiety levels. Patients who were DST non-suppressors were likely to be born during autumn and winter. No effect of the season of birth was found concerning the current severity of suicidal ideation or present or past suicide attempts, although there was a tendency for those patients born during the autumn and winter to manifest a higher rate of suicide attempts. In addition, there was a tendency for those patients born during the winter period to have lower, though not significantly, level of current suicidal ideation.

The theory that *in utero *exposure to flu epidemics might constitute a risk factor for the development of major mental illnesses was developed around 20 years ago, as these epidemics constitute the only reasonable universal factor that fluctuates with season of the year. A study of community data that included patients with schizophrenia or major affective disorders who had given birth to 3 174 children during 1980–1992 in Western Australia suggested that although genetic liability and gene-environment interactions might account for some adverse outcomes, neonatal complications were significantly more likely to occur in winter and low birth weight peaked in spring [12]

Joiner et al. studied 2,514 adolescents and young adults presenting to general practitioners to test whether Australians *in utero *during the Southern hemisphere flu peak would show increased suicidal and depressive symptoms. The results suggested that there was a significant birth-season effect on depressive symptoms and a non-significant similar trend concerning suicidal ideation. Those born in the Southern hemisphere in September to November (analogous to March to May for those born in the Northern hemisphere) showed the highest suicidal and depressive symptoms [13]. These results are remarkably similar to the results of the current study. Similar results were reported in a study of subjects exposed as fetuses to the type A2/Singapore influenza epidemic in greater Helsinki, Finland. That study reported a significant increase in the diagnosis of unipolar major depression for those exposed during their second trimester [3]. Mino et al. collected data from governmental statistics and the Patient Survey in Japan in 1996, and detected 13 969 mood disorder patients. They reported a birth excess of patients from winter to early spring in both sexes, compared to births of the general population with the magnitude of the excess being larger in females than in males [14].

However, there are studies reporting no birth-season effect. Crow and Done searched the data concerning 945 mothers exposed to influenza during their second trimester of gestation and found no increased rates of schizophrenia or affective disorders for the offspring [15]. Two other studies reported that a low affective disorders rate in relationship to influenza exposure *in utero *[16] might be restricted for females [17]. Other authors suggest this decrease is not gender related [18]. In addition, against the prevailing theory concerning the second trimester of gestation, an effect of the first trimester [19] or the first and the third trimesters of gestation are also reported [17].

In the study of Parker et al. [20], information on sex, diagnosis and date of birth were obtained on all 20 358 patients first admitted to psychiatric facilities in New South Wales during a 4-year span. Results showed a significant winter excess for the female schizophrenic group, while a significant spring excess was found for neurotic patients, most marked in those with anxiety neurosis. The authors suggested that the relationship between schizophrenia and winter birth is consequent upon a greater sensitivity of schizophrenics to those physiological factors that determine conception in the general population. Of interest is the finding that 'neurotic' patients are usually born in spring. Similar results were reported by Hare in 1975 [21]. That study was conducted in England and Wales, using patients born between 1921 and 1955, and reported that compared with all live births, manic depression was associated with a significant excess of births in the first quarter, and a corresponding deficiency in the third quarter of the year. In contrast, neurotic depression showed no such association. However results of that study are difficult to interpret because of lack of widely accepted operational diagnostic criteria used. What is more interesting from this study is the lack of any association between season of birth and 'neurotic' depression. In another study on bipolar disorder, results showed that the birth dates of most bipolar I patients showed a tendency to peak during spring and autumn, while bipolar II patients were born mostly in summer and winter [22].

In the study of Brochard et al. in 1994 [23], which is a more recent retrospective study from France, the months of birth of 3 106 psychiatric inpatients were compared to those of 1 943 surgical patients, and to a sample of 10 003 572 births in France between the years 1977 and 1989. Flexible DSM III-R categories were used. Results showed that there was no deviation of neurotic-reactive depressions from the general population, but there was a deviation concerning unipolar major depression, with a significant excess of births during the 'dark' or 'cold' season of the year, especially around the winter solstice. This is to a large extent in accord with the findings of the current study. The bipolar group followed the same tendency, though to a lesser degree and for subjects born before 1940 only. The authors suggested that their results seem to be relevant to the traditional endogenous psychogenic dichotomy, with a 'cold' or 'dark' seasonality of births in the first case, and no particular seasonality in the second case. A second study by D'Amato et al. in 1991 [24], also from France, reported a significant excess of births of schizophrenics for the winter quarter and for the cold half of the year, with the disorganized-type patients making the difference.

However, although the seasonality in the births of certain types of patients suffering from major depression seems to be confirmed by the present study, the etiology of this remains only speculative. Another confounding factor could be the fact that during the 'cold' months (November to February) depressive patients are reported to have lower rates of DST non-suppression as well as lower concentrations of post-dexamethasone plasma cortisol compared to the 'hot months' (March to October), despite similar pre-dexamethasone cortisol levels. This could suggest less disturbance of HPA axis function in winter months in depressed patients [25-27]. However, in the current study no systematic bias is present concerning the season of data collection. DST was performed and data were collected during all seasons of the years. It should also be noted that DST results tend to be the same between consecutive depressive episodes of the same patient; that is, a DST suppressor in the current episode is more likely to be suppressor also in future episodes [28].

A significant point of critique of the literature until today is the fact that most studies are epidemiological and population based, therefore they lack an in-depth evaluation of patients; sometimes their samples can include subjects with high levels of dysphoria instead of depression per se, because depressive scales are relatively non-specific.

The interpretation of the results of the current study is difficult. The relationship of the season of birth to current symptomatology, and the similar but weak relation to suicidal acts, is in accord with the literature. However, interpreting the DST data is far more difficult. The finding that DST non-suppressors are likely to be born almost exclusively (90%) during the autumn and winter cannot be interpreted in a satisfactory way. This is partially because the true meaning of DST non-suppression in depression is elusive. One possible explanation could be based on the assumption that the DST non-suppression represents a compensatory mechanism that increases catecholamine activity in an effort to self-treat depression The international literature supports a minimal correlation between DST and symptomatology [29]. In some patients, the HPA axis can hyperfunction in a compensatory effort to maintain the catecholamine function at normal levels, or alternatively, receptors might get hypersensitive [30]. In this context, these DST non-suppressor patients are less pathologic, suffer from less 'biochemically severe' depression and have a neuroendocrine system that is still flexible and can react to stimuli and possibly to medication. This could be why these patients are considered to be more responsive to antidepressant agents. Of course this theory is in disagreement with the almost 25-year-old traditional view that considers DST suppressors as 'neurotic patients', however it is possible that the mechanisms that affect DST results during transient stressful situations differ from those involved in melancholic depression.

The positive aspects of the current paper are that it is the first study to use modern and rigid diagnostic criteria and diagnostic methodology; the clinical subtypes of depression, though reliable [31], constitute an insufficient approach, especially as there are data available on the existence of biological differences between subtypes [32-34]. It is also the first to use a biological marker (DST) to categorize patients. The negative aspects of the current work are that there is a lack of data concerning DST in controls, and a relatively small size of patient sample.

The main disadvantage is the lack of data concerning DST in controls. However, the authors believe that the reported low percentage of non-suppressors in this group, and the possibility that this is related to family history or susceptibility to develop depression, make the potential influence of this lack of data in the interpretation of results very small.

## Conclusion

The current study confirms the seasonality of birth rate of patients suffering from specific types of depression, and suggests that the clinical picture and course might relate to events during the prenatal or perinatal periods. The finding that depressed patients born during the spring (March/April/May) manifested higher levels of depressive symptomatology while those born during the summer manifested the lowest anxiety levels in addition to the finding that DST non-suppressors were likely to be born during autumn and winter are difficult to interpret and could not be explained on the basis of the virus infection *in utero *alone. Further research is necessary to elucidate this highly complex possible interaction between chronobiology and a neurodevelopmental effect on the development of adult-onset major depression.

## Competing interests

The author(s) declare that they have no competing interests.

## Authors' contributions

KNF designed the study, collected clinical data, analyzed data and wrote the draft paper, AI contributed to the study design and interpretation of data and final formulation of the manuscript, MK contributed to the design of the biochemical factors of the study method, interpretation of data and final formulation of the manuscript, GK and CI contributed to the study design and interpretation of data and final formulation of the manuscript.
